# Is the golden ratio a universal constant for self-replication?

**DOI:** 10.1371/journal.pone.0200601

**Published:** 2018-07-16

**Authors:** Yu Liu, David J. T. Sumpter

**Affiliations:** Department of Mathematics, Uppsala University, Uppsala, Sweden; University of Lincoln, UNITED KINGDOM

## Abstract

The golden ratio, *ϕ* = 1.61803…, has often been found in connection with biological phenomena, ranging from spirals in sunflowers to gene frequency. One example where the golden ratio often arises is in self-replication, having its mathematical origins in Fibonacci’s sequence for “rabbit reproduction”. Recently, it has been claimed that *ϕ* determines the ratio between the number of different nucleobases in human genome. Such empirical examples continue to give credence to the idea that the golden ratio is a universal constant, not only in mathematics but also for biology. In this paper, we employ a general framework for chemically realistic self-replicating reaction systems and investigate whether the ratio of chemical species population follows “universal constants”. We find that many self-replicating systems can be characterised by an algebraic number, which, in some cases, is the golden ratio. However, many other algebraic numbers arise from these systems, and some of them—such as $\sqrt[{3}]{2}=1.25992\ldots$ and 1.22074… which is also known as the *3rd lower golden ratio*—arise more frequently in self-replicating systems than the golden ratio. The “universal constants” in these systems arise as roots of a limited number of distinct characteristic equations. In addition, these “universal constants” are transient behaviours of self-replicating systems, corresponding to the scenario that the resource inside the system is infinite, which is not always the case in practice. Therefore, we argue that the golden ratio should not be considered as a special universal constant in self-replicating systems, and that the ratios between different chemical species only go to certain numbers under some idealised scenarios.

## Introduction

The golden ratio $(1+\sqrt{5})/2=1.61803\ldots$, usually denoted by the Greek letter *ϕ* (PHI), has attracted broad attention for a long time, with suggestions that they are aesthetically significant and ubiquitous in nature. These claims were made particularly prominent in Dan Brown’s book (and later film) *The Da Vinci Code*: (quote) *Despite PHI’s seemingly mystical mathematical origins, Langdon explained, the truly mind-boggling aspect of PHI was its role as a fundamental building block in nature. Plants, animals, and even human beings all possessed dimensional properties that adhered with eerie exactitude to the ratio of PHI to 1… “PHI’s ubiquity in nature,” Langdon said, killing the lights, “clearly exceeds coincidence, and so the ancients assumed the number PHI must have been preordained by the Creator of the universe…”* [1]

The basis of Dan Brown’s fictional discussion of the golden ratio *ϕ* originated from “scientific” studies (as documented by paper [2] and [3]). For example, the Great Pyramid of Khufu was claimed to be designed by ancient Egyptians to manifest *ϕ*, i.e., the ratio of the slant height to half of the length of the base was designed to be *ϕ*. Moreover, nautilus shell was believed to be a logarithmic spiral whose growth factor is *ϕ*. Other examples include the design of the Parthenon, Leonardo da Vinci’s paintings, the ratios in a human body, lengths of passages in the Aeneid.

These claims are, however, mostly false or misleading [2–4]. For example, although today the ratio of the slant height to half of the length of the base of the Great Pyramid happened to be *ϕ* approximately, the height was quite different when it was built over four thousand years ago, and there is no evidence that ancient Egyptians even knew the golden ratio [2]. A field study by Falbo showed that nautilus shell is quite far from a golden spiral [3]. For the Parthenon, there are many different numbers of its dimensions in the literature (probably because measurements are made between different points by different people), allowing a golden ratio enthusiast to choose whatever numbers to fit *ϕ* [2]. But sadly, despite the dubious nature of the claims, the golden ratio even remains a feature of some school textbooks, such as [5].

One area of biology in which the golden ratio has a genuine role is in phyllotaxis, i.e., the arrangement of leaves on the stem of a plant. The seeds in the head of a ripe sunflower fit neatly together into two interlocking families of helical spirals. The number of clockwise and anti-clockwise winding spirals are often both successive numbers of the Fibonacci sequence [6]. For example, a sunflower might have 34 clockwise spirals and 21 anti-clockwise spirals, where 34 and 21 are two successive numbers in the Fibonacci sequence, i.e., 1, 1, 2, 3, 5, 8, 13, 21, 34, 55, 89, 144, …, which satisfies

$$\begin{array}{c} X_{t}=X_{t-1}+X_{t-2} \end{array}$$ (1)

The limit of the ratio between two successive Fibonacci numbers is the golden ratio, namely, lim_*t*→∞_ *X*_*t*_/*X*_*t*−1_ = *ϕ*. These sequences are genuine biological phenomenon, not just in sunflowers, but in plants such as pinecones, aloe, pineapples, and cacti [6–8]. It should be stressed however that non-Fibonacci phyllotaxy is also abundant [7, 9]. Several theories have been proposed to explain the biochemical and mechanical mechanisms for these patterns (reviewed by paper [10]), including Alan Turing’s morphogenesis theory [11, 12].

The Fibonacci process Eq (1) corresponds to self-replicating processes, having its origin in the growth pattern of the “Fibonacci rabbit”, an idealised scenario first considered by the Italian mathematician Fibonacci in his book *Liber Abaci* (1202). The idea is as follows: rabbits never die; it takes one month for a pair of infant rabbits to become a pair of adults; an adult pair always gives birth to an infant pair; the system starts with one pair of adult rabbits. This gives rise to Eq (1), where *X*_*t*_ is the number of adult pairs at month *t*, and the number of infant pairs at month *t* is *X*_*t*−1_. So the ratio of the number of adult pairs over the number of infant pairs goes to *ϕ* as *t* → ∞.

Recently, the golden ratio was suggested to appear in another totally different field: the human genome. Dress *et al*. proposed that the growth pattern of repetitive DNAs is analogous to the pattern described by the Fibonacci process Eq (1) (repetitive DNAs are those built from a basic short DNA sequence that is repeated many times, often referred to as “junk DNA” and accounted for a large fraction of the whole human genome) [13]. Yamagishi and Shimabukuro argued that the distribution of the values of *P*_*A*_ + *P*_*C*_ for the 24 human chromosomes can also be explained by the Fibonacci process Eq (1) where *P*_*A*_ is the frequency of codon A and likewise, *P*_*C*_ is the frequency of codon C [14]. Moreover, Perez calculated that in the single-stranded whole human DNA, (*P*_*C*_ + *P*_*G*_)/(*P*_*A*_ *P*_*T*_) is approximately (3 − *ϕ*)/2 [15]. These empirical examples continue to give credence to the idea that *ϕ* is a universal constant, not only in mathematics but also for biology.

Another famous model that can generate *ϕ* is the L-system (a type of formal grammar) [16]. The specific L-system that generates *ϕ* has the following rules: for each generation, each Q becomes a Q and an S, while each S becomes a Q. The system is initialised with a string composed of only one Q. So at the first generation, the string becomes QS; at the second generation, the string becomes QSQ; then QSQQS, QSQQSQSQ, … The number of Q at each generation forms the Fibonacci sequence (as well as the number of S but with one generation lag). So the ratio of the number of Q over the number of S goes to *ϕ*. This specific L-system can actually be written as the following “reaction system”,

$$\{\begin{array}{ll} \text{Q} & \to \text{Q}+\text{S}\\ \text{S} & \to \text{Q} \end{array}$$ (2)

This process is equivalent to the Fibonacci rabbit model. Note that Eq (2) also involves self-replication, because the two reactions add up to Q → Q + Q (by cancelling S on both sides) and Q is thus self-replicating. Similarly, the genealogical tree model [17, 18] which can also generate *ϕ* has the same argument.

These models do, from a mathematical viewpoint, give rise to the golden ratio, but our question is whether the golden ratio has a special role in natural self-replicating systems in general, as implied by the authors in papers [13–15]. Should we expect, in a wide range of different self-replicating systems, to find that the ratio of different components in the system is the golden ratio? And if not, are there other important mathematical constants that we should expect to see in connection to self-replicating systems?

To answer these questions we employ a framework of chemically realistic self-replicating reaction systems, introduced by us in paper [19]. This theory has practical significance to real chemistry and biology, and instantiations of this framework can capture a wide range of different natural chemical systems such as the citric acid cycle and the *formose* reaction. In this theory, a self-replicating system consists of a few chemical reactions, and self-replication is a system-level property: each reaction in the system is not able to self-replicate but the system self-replicates as a whole. If the golden ratio *ϕ* does have a special role in natural self-replicating systems in general, we would expect to see *ϕ* appears quite a lot in these systems.

This paper is organised as follows. Section *Materials and methods* reintroduces the framework in paper [19] for chemically realistic self-replicating reaction systems. The first part of Section *Results* elaborates on the scheme for population dynamics that we apply on self-replicating systems, and describe two self-replicating systems that are characterised by *ϕ*. In the second part of Section *Results*, we show that all of the analysable self-replicating systems up to a certain size are characterised by a limited number of distinct characteristic equations and numbers. In the third part of Section *Results*, we introduce the population dynamics for chemical reaction systems under the law of mass action, and find that the characteristic number of a self-replicating system is a transient behaviour corresponding to the scenario that the resource inside the system is infinite, which is not always the case in practice.

## Materials and methods

### Chemically realistic self-replicating reaction systems

In an earlier paper [19], we described a general framework to construct chemically realistic reaction systems. In this framework, a molecule is defined by its integer “mass”, *i*, and thus denoted $\bar{i}$; all reactions that conserve mass—the total mass on the reactant side adds up to those on the product side—are possible; only two types of reaction are allowed, synthesis of two molecules to create a molecule of greater mass (e.g., $\bar{2}+\bar{4}\to \bar{6}$) and decomposition into two molecules to create two molecules of lower mass (e.g., $\bar{6}\to \bar{1}+\bar{5}$); every reaction is chemically spontaneous. The last requirement constrains the details of the thermodynamic free energy of each molecule and reaction, which we do not need to consider in this paper, but it does have two consequences related to this paper. The first one is that a reaction and its reverse reaction, e.g., $\bar{2}+\bar{4}\to \bar{6}$ and $\bar{6}\to \bar{2}+\bar{4}$, cannot both appear in one system. The second consequence will be introduced in the next paragraph.

By choosing some reactions that satisfy the requirements above, we can construct a chemically realistic reaction system, namely, an instantiation of the general framework. The following system is one of such instantiations,

$$\begin{array}{c} \left\{ \begin{array}{c} \begin{array}{cc} \bar{2} & \to \bar{1}+\bar{1}\\ \bar{3} & \to \bar{1}+\bar{2}\\ \bar{1}+\bar{4} & \to \bar{5}\\ \bar{5} & \to \bar{2}+\bar{3} \end{array} \end{array}\right. \end{array}$$ (3)

We define the *resource molecule* to be the molecule that only appears on the reactant side ($\bar{4}$ in this case). For any chemically realistic self-replicating system, there is at least one type of resource molecule. This is the second consequence of the “chemical spontaneity” requirement above. We define an *intermediate molecule* to be any molecule that appears on both the reactant side and the product side ($\bar{1}$, $\bar{2}$, $\bar{3}$ and $\bar{5}$ in this case), and the *waste molecule* to be the molecule that only appears on the product side (there is no in this case).

In paper [19], we show that a chemically realistic reaction system is self-replicating if the following three criteria are satisfied:

1 For every reaction, at least one type of its reactants comes from the products of other reactions;
2 There is at least one intermediate molecule that appears on the reactant side fewer times than that on the product side;
3 There are no intermediate molecules that appear on the reactant side more often than on the product side.

In this paper we introduce an alternative, narrower criterion under which certain chemically realistic self-replicating systems are analysable. We say that a self-replicating system is analysable if it satisfies criterion 1, 2, and the following criterion 3*:

3* On the reactant side of the whole system, each intermediate molecule species appears at most once.

Note that system Eq (3) satisfies all of criterion 1, 2 and 3*, and thus is an analysable self-replicating chemical reaction system.

## Results

### Systems characterised by the golden ratio *ϕ*

We now introduce a scheme for population dynamics for analysable self-replicating chemical reaction systems, using system Eq (3) as an example. Let *N*_*i*_(*t*) be the population of molecule species $\bar{i}$ at *generation* *t*. We initialise the system with *N*_1_(0) = *N*_2_(0) = *N*_3_(0) = *N*_5_(0) = 1, i.e., one of each intermediate molecules. In addition, we assume that there is an infinite number of resource molecules $\bar{4}$ inside the system, namely *N*_4_(*t*) = ∞ for all *t*.

We update molecule populations at *generation* *t* + 1 from *t* as follows: for each molecule species $\bar{i}$, find the unique reaction that has $\bar{i}$ on the reactant side; and all of molecules $\bar{i}$ at generation *t*—namely *N*_*i*_(*t*) of $\bar{i}$—are transformed into the products of this corresponding reaction. The criterion 3* above guarantees that there is only one unique reaction that has molecule $\bar{i}$ on the reactant side.

Consider how we apply this updating procedure to system Eq (3):

- Firstly, update *N*_*i*_(0) to obtain *N*_*i*_(1). For molecule $\bar{1}$, due to the fact that the third reaction is the only reaction that has molecule $\bar{1}$ on the reactant side and *N*_1_(0) = 1, we let this one of $\bar{1}$ transform into one of $\bar{5}$ through the third reaction. For molecule $\bar{2}$, due to the fact that the first reaction is the only reaction that has molecule $\bar{2}$ on the reactant side and *N*_2_(0) = 1, we let this one of $\bar{2}$ transform into two of $\bar{1}$ through the first reaction. For molecule $\bar{3}$, because the second reaction is the unique reaction and *N*_3_(0) = 1, we let this one of $\bar{3}$ transform into one of $\bar{1}$ and one of $\bar{2}$. For molecule $\bar{5}$, because the fourth reaction is the unique reaction and *N*_5_(0) = 1, we let this one of $\bar{5}$ transform into one of $\bar{2}$ and one of $\bar{3}$. Therefore, at generation *t* = 1, we have *N*_1_(1) = 3, *N*_2_(1) = 2, *N*_3_(1) = 1 and *N*_5_(1) = 1.
- Then, update *N*_*i*_(1) to obtain *N*_*i*_(2). *N*_1_(1) = 3 of molecule $\bar{1}$ are transformed into three of $\bar{5}$ through the third reaction; *N*_2_(1) = 2 of $\bar{2}$ are transformed into four of $\bar{1}$ through the first reaction; *N*_3_(1) = 1 of $\bar{3}$ is transformed into one $\bar{1}$ and one $\bar{2}$ through the second reaction; and *N*_5_(1) = 1 of $\bar{5}$ is transformed into one $\bar{2}$ and one $\bar{3}$ through the fourth reaction. Therefore, at generation *t* = 2, we have *N*_1_(2) = 5, *N*_2_(2) = 2, *N*_3_(2) = 1 and *N*_5_(2) = 3.
- Similarly, at generation *t* = 3, we have *N*_1_(3) = 5, *N*_2_(3) = 4, *N*_3_(3) = 3 and *N*_5_(3) = 5.
- Continue this updating procedure. Finally we obtain a sequence of *N*_1_(*t*), *N*_2_(*t*), *N*_3_(*t*) and *N*_5_(*t*).

Table 1 shows the result over six generations. Note that this scheme can be applied to any analysable self-replicating system, through the following steps in general: (1) assume an infinite number of resource molecules inside the system; (2) initialise the system with one of each intermediate molecule; and (3) update molecule populations by generations to obtain sequences of molecule populations. This scheme defines a particular population dynamics for self-replicating systems.

**Table 1:** Molecule populations *N*_*i*_(*t*) for the self-replicating chemical reaction system Eq (3) at generation *t*.

Generation *t*	*N*_1_(*t*)	*N*_2_(*t*)	*N*_3_(*t*)	*N*_5_(*t*)
0	1	1	1	1
1	3	2	1	1
2	5	2	1	3
3	5	4	3	5
4	11	8	5	5
5	21	10	5	11
6	25	16	11	21
⋮				⋮

Note that *N*_*i*_(*t* + 1) only depends on *N*_*i*_(*t*), so we can derive a recurrence relation (written as a matrix form):

$$\begin{array}{c} \left(\begin{array}{c} N_{1}\left(t+1\right)\\ N_{2}\left(t+1\right)\\ N_{3}\left(t+1\right)\\ N_{5}\left(t+1\right) \end{array})=(\begin{array}{cccc} 0 & 2 & 1 & 0\\ 0 & 0 & 1 & 1\\ 0 & 0 & 0 & 1\\ 1 & 0 & 0 & 0 \end{array})(\begin{array}{c} N_{1}\left(t\right)\\ N_{2}\left(t\right)\\ N_{3}\left(t\right)\\ N_{5}\left(t\right) \end{array}\right) \end{array}$$ (4)

We call this *m* × *m* matrix as the *recurrence matrix*, denoted as **A**, where *m* is the number of intermediate molecules. Denote the vector of molecule populations (*N*_1_(*t*), *N*_2_(*t*), *N*_3_(*t*), *N*_5_(*t*))^⊺^ as **N**(*t*), then Eq (4) becomes

$$\begin{array}{c} N(t+1)=AN(t) \end{array}$$ (5)

Therefore, we can also use **A** to calculate the population sequences:

$$\begin{array}{c} N\left(t\right)=A^{t}\;N\left(0\right) \end{array}$$ (6)

Eqs (5) and (6) also represent this particular population dynamics in general.

**Fig 1:**
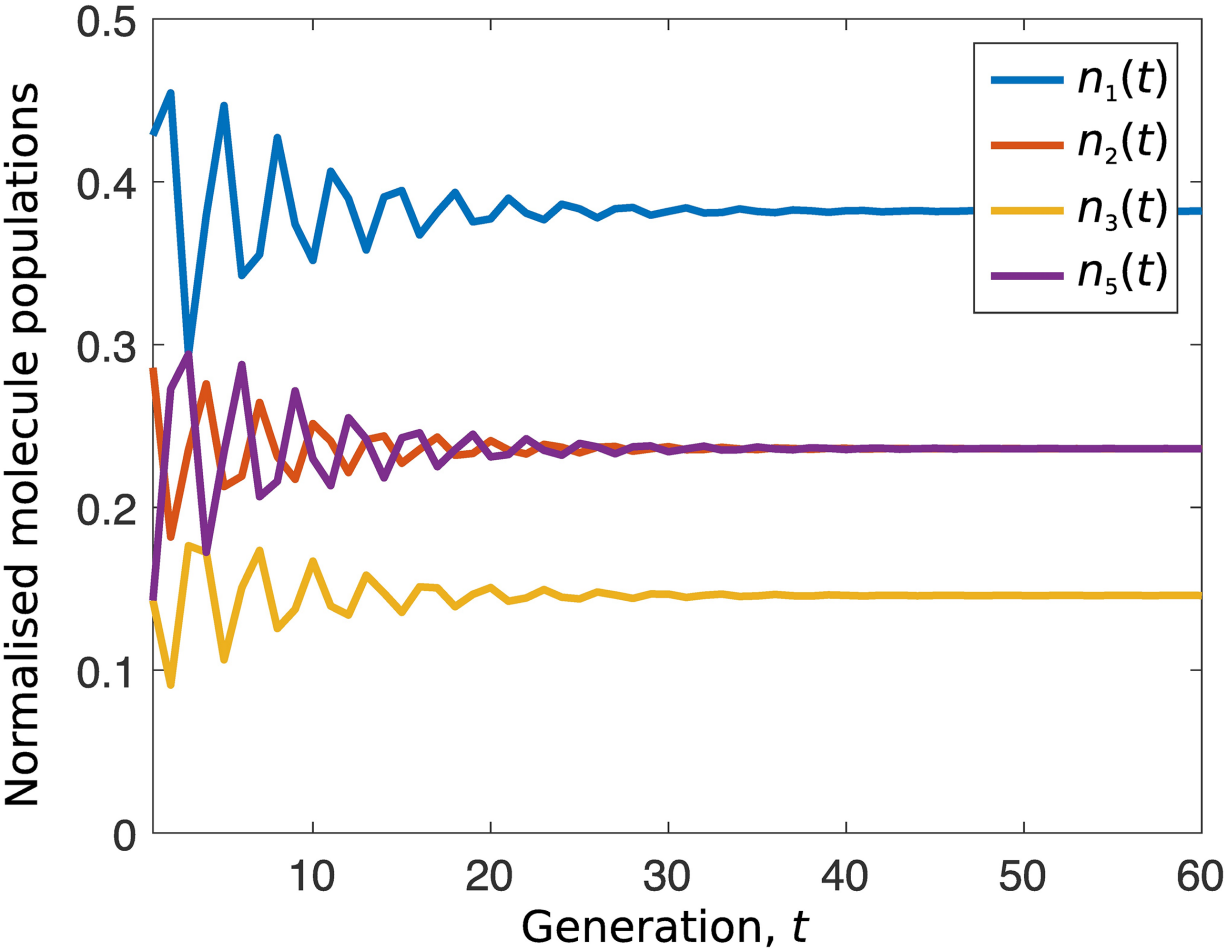
Normalised molecule population *n*_*i*_(*t*) = *N*_*i*_(*t*)/∑_*l*_ *N*_*l*_(*t*) for 60 generations for the chemically realistic self-replicating reaction system Eq (3). Specifically, lim_*t*→∞_ *n*_1_(*t*) = 0.38196…, lim_*t*→∞_ *n*_2_(*t*) = lim_*t*→∞_ *n*_5_(*t*) = 0.23606… and lim_*t*→∞_ *n*_3_(*t*) = 0.14589…

Through either of the steps above, we obtain the sequences *N*_*i*_(*t*). We further find that the normalised population of each molecule species saturates, as shown in Fig 1, and that

$$\begin{array}{c} \lim_{t\to \infty }\frac{N_{1}\left(t\right)}{N_{2}\left(t\right)}=1.61803\ldots =\phi \end{array}$$ (7)

i.e., the ratio of populations of molecule $\bar{1}$ over that of $\bar{2}$ goes to the golden ratio as *t* → ∞.

We now rigorously prove Eq (7). Based on Eq (4), we have *N*_5_(*t* + 1) = *N*_1_(*t*), so *N*_3_(*t* + 1) = *N*_5_(*t*) = *N*_1_(*t* − 1), and then we have *N*_2_(*t* + 1) = *N*_3_(*t*) + *N*_5_(*t*) = *N*_1_(*t* − 2) + *N*_1_(*t* − 1). Finally,

$$\begin{array}{ll} N_{1}\left(t+1\right) & =2N_{2}\left(t\right)+N_{3}\left(t\right)=2\left(N_{1}\left(t-3\right)+N_{1}\left(t-2\right)\right)+N_{1}\left(t-2\right)\\ & =3N_{1}\left(t-2\right)+2N_{1}\left(t-3\right)\\ \Leftrightarrow N_{1}\left(t\right) & =3N_{1}\left(t-3\right)+2N_{1}\left(t-4\right) \end{array}$$

Based on standard skills for solving recursive sequences [20], the characteristic equation of the sequence *N*_1_(*t*) is

$$\begin{array}{c} \lambda^{4}-3\lambda -2=\left(\lambda^{2}-\lambda -1\right)\left(\lambda^{2}+\lambda +2\right)=0 \end{array}$$ (8)

and its four roots are

$$\begin{array}{c} \left\{ \begin{array}{c} \begin{array}{cc} \lambda_{1} & =(1+\sqrt{5})/2=\phi \\ \lambda_{2} & =(1-\sqrt{5})/2\\ \lambda_{3} & =\left(-1+\sqrt{7}j\right)/2\approx \sqrt{2}e^{1.93j}\\ \lambda_{4} & =\left(-1-\sqrt{7}j\right)/2\approx \sqrt{2}e^{-1.93j} \end{array} \end{array}\right. \end{array}$$ (9)

where *j* is the unit imaginary number. Therefore, the closed form of this sequence is $N_{1}\left(t\right)=\alpha \lambda_{1}^{t}+\beta \lambda_{2}^{t}+\delta \lambda_{3}^{t}+\eta \lambda_{4}^{t}$ where *α*, *β*, *δ* and *η* are constants determined by the initial value *N*_1_(0) [20]. Note that the absolute value of λ_1_ is the largest among all roots, resulting in $\lim_{t\to \infty }\lambda_{2}^{t}/\lambda_{1}^{t}=0$, and the same argument for λ_3_ and λ_4_. Therefore, we have

$$\begin{array}{cc} \lim_{t\to \infty }\frac{n_{1}\left(t\right)}{n_{2}\left(t\right)}= & \lim_{t\to \infty }\frac{N_{1}\left(t\right)}{N_{2}\left(t\right)}=\lim_{t\to \infty }\frac{N_{1}\left(t\right)}{N_{1}\left(t-3\right)+N_{1}\left(t-2\right)}\\ = & \lim_{t\to \infty }\frac{\alpha \lambda_{1}^{t}+\beta \lambda_{2}^{t}+\delta \lambda_{3}^{t}+\eta \lambda_{4}^{t}}{\alpha \lambda_{1}^{t-3}+\beta \lambda_{2}^{t-3}+\delta \lambda_{3}^{t-3}+\eta \lambda_{4}^{t-3}+\alpha \lambda_{1}^{t-2}+\beta \lambda_{2}^{t-2}+\delta \lambda_{3}^{t-2}+\eta \lambda_{4}^{t-2}}\\ = & \lim_{t\to \infty }\frac{1+\frac{\beta \lambda_{2}^{t}}{\alpha \lambda_{1}^{t}}+\frac{\delta \lambda_{3}^{t}}{\alpha \lambda_{1}^{t}}+\frac{\eta \lambda_{4}^{t}}{\alpha \lambda_{1}^{t}}}{\frac{1}{\lambda_{1}^{3}}+\frac{\beta \lambda_{2}^{t-3}}{\alpha \lambda_{1}^{t}}+\frac{\delta \lambda_{3}^{t-3}}{\alpha \lambda_{1}^{t}}+\frac{\eta \lambda_{4}^{t-3}}{\alpha \lambda_{1}^{t}}+\frac{1}{\lambda_{1}^{2}}+\frac{\beta \lambda_{2}^{t-2}}{\alpha \lambda_{1}^{t}}+\frac{\delta \lambda_{3}^{t-2}}{\alpha \lambda_{1}^{t}}+\frac{\eta \lambda_{4}^{t-2}}{\alpha \lambda_{1}^{t}}}\\ = & \frac{1}{\lambda_{1}^{-3}+\lambda_{1}^{-2}}=\frac{1+\sqrt{5}}{2}=\phi \end{array}$$

Note that Eq (8) is also the characteristic equation of the matrix **A** in Eq (4), and the four roots in Eq (9) are thus the eigenvalues of **A**. Therefore, the recurrence matrix **A** gives all the information of the sequence. Note that the largest absolute value among **A**’s eigenvalues corresponds to *ϕ*. We call this largest eigenvalue the *characteristic number*, denoted Λ, of the self-replicating system.

Now we show another example of a chemically realistic self-replicating reaction system that is characterised by *ϕ*,

$$\begin{array}{c} \left\{ \begin{array}{c} \begin{array}{cc} \bar{3} & \to \bar{1}+\bar{2}\\ \bar{4} & \to \bar{1}+\bar{3}\\ \bar{1}+\bar{6} & \to \bar{7}\\ \bar{2}+\bar{5} & \to \bar{7}\\ \bar{7} & \to \bar{3}+\bar{4} \end{array} \end{array}\right. \end{array}$$ (10)

where the resource molecules are $\bar{5}$ and $\bar{6}$. It has the recurrence relation

$$\left(\begin{array}{c} N_{1}\left(t+1\right)\\ N_{2}\left(t+1\right)\\ N_{3}\left(t+1\right)\\ N_{4}\left(t+1\right)\\ N_{7}\left(t+1\right) \end{array})=(\begin{array}{ccccc} 0 & 0 & 1 & 1 & 0\\ 0 & 0 & 1 & 0 & 0\\ 0 & 0 & 0 & 1 & 1\\ 0 & 0 & 0 & 0 & 1\\ 1 & 1 & 0 & 0 & 0 \end{array})(\begin{array}{c} N_{1}\left(t\right)\\ N_{2}\left(t\right)\\ N_{3}\left(t\right)\\ N_{4}\left(t\right)\\ N_{7}\left(t\right) \end{array}\right)$$

The characteristic equation of the recurrence matrix is

$$\begin{array}{c} \lambda \left(\lambda^{4}-3\lambda -2\right)=\lambda \left(\lambda^{2}-\lambda -1\right)\left(\lambda^{2}+\lambda +2\right)=0 \end{array}$$

This equation has five roots, where four of them are the same as in Eq (9) and another one is 0. Therefore, the characteristic number Λ for this system is also *ϕ*. The golden ratio *ϕ* occurs when the characteristic equation has the factor (λ^2^ − λ − 1). We have now seen that this occurs for at least two analysable self-replicating systems.

Let us now consider the L-system (equivalently, the Fibonacci rabbit model) given in Eq (2). This system satisfies criterion 1, 2 and 3*, and therefore we can employ the scheme for population dynamics defined at the start of this section, namely Eq (5). Specifically, we obtain the recurrence relation:

$$\left(\begin{array}{c} N_{Q}\left(t+1\right)\\ N_{S}\left(t+1\right) \end{array})=(\begin{array}{cc} 1 & 1\\ 1 & 0 \end{array})(\begin{array}{c} N_{Q}\left(t\right)\\ N_{S}\left(t\right) \end{array}\right)$$

The characteristic equation for this recurrence matrix is λ^2^ − λ − 1 = 0. So the characteristic number Λ for this system is *ϕ*, thus recovering the golden ratio usually associated with the L-system and the Fibonacci rabbit model.

There is however a serious problem with the biological interpretation of system Eq (2): it is not a chemically realistic reaction system. Because Eq (2) does not fulfil the requirements mentioned at the start of this section to be chemically realistic, e.g., mass cannot be conserved in the reaction Q → Q + S, as S is produced out of nothing. The intermediate steps for producing S and Q are not fully described.

We can, nonetheless, construct a chemically realistic self-replicating system which is analogous to Eq (2). The following system is one (non-unique) example:

$$\begin{array}{c} \left\{ \begin{array}{c} \begin{array}{cc} \bar{1}+\bar{4} & \to \bar{5}\\ \bar{11} & \to \bar{1}+\bar{10}\\ \bar{10} & \to \bar{3}+\bar{7}\\ \bar{5}+\bar{6} & \to \bar{11}\\ \bar{3} & \to \bar{1}+\bar{2} \end{array} \end{array}\right. \end{array}$$ (11)

After omitting to write the resource ($\bar{4}$ and $\bar{6}$) and the waste ($\bar{2}$ and $\bar{7}$), the first four reactions add up to $\bar{1}\to \bar{1}+\bar{3}$ (by cancelling $\bar{5}$, $\bar{10}$ and $\bar{11}$ on both sides), and the last reaction becomes $\bar{3}\to \bar{1}$. These two reactions are identical to Eq (2) as $\bar{1}$ corresponds to Q and $\bar{3}$ corresponds to S. However, the characteristic number Λ for system Eq (11) is 1.19385…, which is not *ϕ*. Thus we cannot reliably use the L-system to explain why the golden ratio appears in some biological systems.

### Distribution of the characteristic number Λ

**Fig 2:**
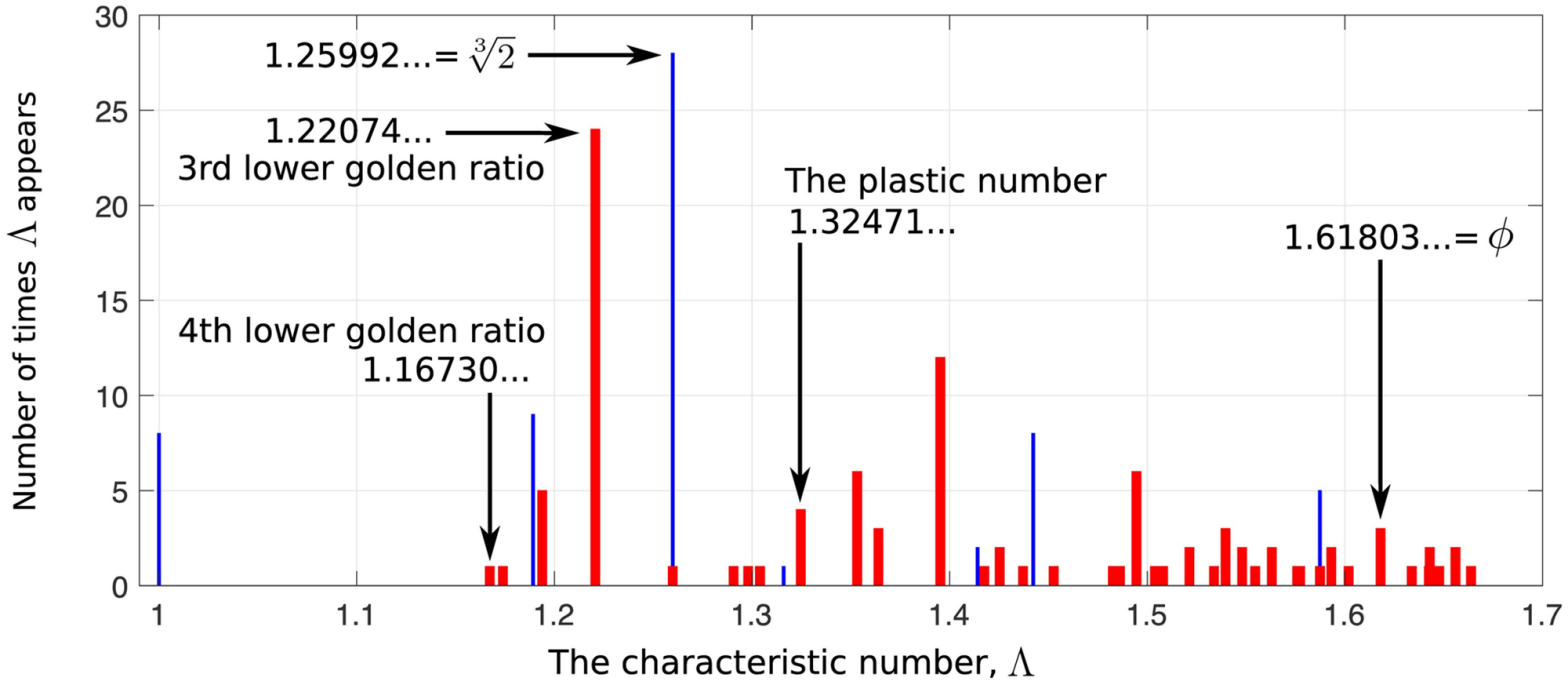
Distribution of the characteristic numbers Λ for all the analysable self-replicating chemical reaction systems up to where the possibly largest molecule is $\bar{7}$. The blue lines correspond to all *integer self-replicating systems*, while the red bars correspond to all other systems.

We now investigate a larger range of chemically realistic self-replicating reaction systems in order to determine which numbers typically characterise their behaviour. We consider all of the analysable self-replicating systems up to where the possibly largest molecule is $\bar{7}$. We exhaustively checked every possible reaction system up to $\bar{7}$ to see whether it satisfies criterion 1, 2 and 3* in Section *Materials and methods*. Then we found 162 analysable self-replicating systems in total (currently we cannot go beyond $\bar{7}$ because the number of possible reaction systems increases so fast that the computation time becomes too long). The distribution of the characteristic numbers Λ for all of them is shown in Fig 2.

The most frequent characteristic number is $\Lambda =1.25992\ldots =\sqrt[{3}]{2}$, which appeared 28 times. The following system Eq (12) is one such example,

$$\begin{array}{c} \left\{ \begin{array}{c} \begin{array}{cc} \bar{1}+\bar{2} & \to \bar{3}\\ \bar{1}+\bar{3} & \to \bar{4}\\ \bar{4} & \to \bar{2}+\bar{2} \end{array} \end{array}\right. \end{array}$$ (12)

where molecule $\bar{1}$ is the resource. We have the recurrence relation

$$\begin{array}{c} \left(\begin{array}{c} N_{2}\left(t+1\right)\\ N_{3}\left(t+1\right)\\ N_{4}\left(t+1\right) \end{array})=(\begin{array}{ccc} 0 & 0 & 2\\ 1 & 0 & 0\\ 0 & 1 & 0 \end{array})(\begin{array}{c} N_{2}\left(t\right)\\ N_{3}\left(t\right)\\ N_{4}\left(t\right) \end{array}\right) \end{array}$$ (13)

where the 3 × 3 matrix is the recurrence matrix **A**. Table 2 shows *N*_*i*_(*t*) for several generations. We find that the population of any molecule species doubles every three generations, i.e.,

$$N_{i}\left(t+3\right)=2N_{i}\left(t\right)\;\;\text{holds}\;\text{for}\;\text{any}\;t\text{,}\;\text{and}\;i=2,3,4.$$

**Table 2:** Molecule populations *N*_*i*_(*t*) for the self-replicating chemical reaction system Eq (12) at generation *t*.

Generation *t*	*N*_2_(*t*)	*N*_3_(*t*)	*N*_4_(*t*)
0	1	1	1
1	2	1	1
2	2	2	1
3	2	2	2
4	4	2	2
5	4	4	2
6	4	4	4
⋮			⋮

Equivalently, **N**(*t* + 3) = 2**N**(*t*) holds for any *t*, where **N**(*t*) = (*N*_2_(*t*), *N*_3_(*t*), *N*_4_(*t*))^⊺^. We call this type of system as the *integer self-replicating system*, namely, those satisfying that

$$N\left(t+p\right)=A^{p}\;N\left(t\right)=RN\left(t\right)\;\;\text{holds}\;\text{for}\;\text{any}\;t,$$

where **N**(*t*) is the *m* × 1 vector for populations of intermediate molecules at generation *t*, the *period* *p* is a positive integer, and the *growth rate* *R* is a positive integer. We further find that all of the eigenvalues of **A** in Eq (13) have the same absolute value and this value is $\sqrt[{p}]{R}$ ($\sqrt[{3}]{2}$ in this case), which also means that the characteristic number $\Lambda =\sqrt[{p}]{R}$. It is a special property compared with other systems such as Eqs (3) and (10) where the absolute values of the eigenvalues of their corresponding recurrence matrix are not all equal.

System Eq (12) is just one specific integer self-replicating system. The blue lines in Fig 2 represent all integer self-replicating systems, which are quite common. Their characteristic numbers, from small to large, are $1,\sqrt[{4}]{2},\sqrt[{3}]{2},\sqrt[{4}]{3},\sqrt[{4}]{4},\sqrt[{3}]{3}$, and $\sqrt[{3}]{4}$, respectively.

The second most frequent characteristic number in Fig 2 is Λ = 1.22074… This is known as the 3*rd lower golden ratio*, because of the fact that Λ^3^ = 1 + 1/Λ. In general, the number *x* that satisfies *x*^*k*^ = 1 + 1/*x* where *k* is an integer is proposed to be the *kth lower golden ratio* [21]. The following system is one example system characterised by the 3rd lower golden ratio.

$$\left\{ \begin{array}{c} \begin{array}{cc} \bar{3} & \to \bar{1}+\bar{2}\\ \bar{4} & \to \bar{1}+\bar{3}\\ \bar{1}+\bar{5} & \to \bar{6}\\ \bar{6} & \to \bar{2}+\bar{4} \end{array} \end{array}\right.$$

where molecule $\bar{5}$ is the resource. It has the recurrence relation

$$\left(\begin{array}{c} N_{1}\left(t+1\right)\\ N_{3}\left(t+1\right)\\ N_{4}\left(t+1\right)\\ N_{6}\left(t+1\right) \end{array})=(\begin{array}{cccc} 0 & 1 & 1 & 0\\ 0 & 0 & 1 & 0\\ 0 & 0 & 0 & 1\\ 1 & 0 & 0 & 0 \end{array})(\begin{array}{c} N_{1}\left(t\right)\\ N_{3}\left(t\right)\\ N_{4}\left(t\right)\\ N_{6}\left(t\right) \end{array}\right)$$

The eigenvalues of the recurrence matrix are 1.22074…, −0.24812… ± *j*1.03398…, and −0.72449…, respectively.

Note that the 1st lower golden ratio (namely, *ϕ*) appears three times; the 2nd lower golden ratio 1.32471… (also called the *plastic number*) appears four times; and the 4th lower golden ratio 1.16730… appears once (Fig 2).

In order to explain why certain characteristic numbers arise, we note that the *m* × *m* recurrrence matrix **A** = (*a*_*jk*_) for any analysable self-replicating chemical reaction system must satisfy the following conditions:

1. It is a non-negative integer square matrix. This is because any reaction involves only integer number of molecules.
2. All entries on the main diagonal are 0, namely *a*_*jj*_ = 0. This is because no molecule can appear on both sides of a reaction (e.g., $\bar{2}+\bar{3}\to \bar{2}$ is not allowed, since the mass has to be conserved).
3. *a*_*jk*_ and *a*_*kj*_ cannot be both larger than 0, that is, *a*_*jk*_ ⋅ *a*_*kj*_ = 0. This is because a reaction and its reverse reaction cannot both appear in one system.
4. The sum of any column is either 0, 1 or 2. This is because of criterion 3* in Section *Materials and methods* and also because each reaction produces no more than two molecules.
5. The sum of at least one column is 2. This is because of criterion 2 in Section *Materials and methods*.
6. The sum of any row is at least 1. This is because of criterion 1 in Section *Materials and methods*.

Therefore, the characteristic equation of **A** has the general form (referring to S1 Appendix for the derivation):

$$\lambda^{m}-\sigma_{3}\lambda^{m-3}+\sigma_{4}\lambda^{m-4}-\cdots +{\left(-1\right)}^{m-1}\sigma_{m-1}\lambda +{\left(-1\right)}^{m}\sigma_{m}=0$$

where *σ*_3_, *σ*_4_, ⋯, *σ*_*m*_ are all integers. Note that (1) this polynomial is always monic, i.e., the leading coefficient is 1; (2) the term λ^*m*−1^ is zero because of condition 2 of **A** mentioned above; (3) the term λ^*m*−2^ is zero because of condition 3 of **A** mentioned above.

For all of the chemically realistic self-replicating systems we investigated (where the largest molecule is $\bar{7}$), there are at most six types of intermediate molecules (since at least one molecule species must be the resource). Therefore, we only have four cases: For any 3-intermediate-molecule system, the characteristic equation is

$$\begin{array}{c} \lambda^{3}-\det\left(A\right)=0 \end{array}$$ (14)

For any 4-intermediate-molecule system, the characteristic equation is

$$\lambda^{4}-\sigma_{3}\lambda +\det\left(A\right)=0$$

For any 5-intermediate-molecule system, the characteristic equation is

$$\lambda^{5}-\sigma_{3}\lambda^{2}+\sigma_{4}\lambda -\det\left(A\right)=0$$

For any 6-intermediate-molecule system, the characteristic equation is

$$\lambda^{6}-\sigma_{3}\lambda^{3}+\sigma_{4}\lambda^{2}-\sigma_{5}\lambda +\det\left(A\right)=0$$

Here we list four properties of the characteristic number Λ for self-replicating systems:

1. Any Λ appeared in Fig 2 is the largest roots of either of the four characteristic equations above, which also means that any Λ is an *algebraic number*.
2. The self-replicating systems that is characterised by the golden ratio *ϕ* have at least four types of intermediate molecules.
3. From Eq (14), we see that for any 3-intermediate-molecule system, the absolute values of all of **A**’s eigenvalues are equal, and thus $\Lambda =\sqrt[{3}]{\det(A)}$. Therefore, any 3-intermediate-molecule self-replicating system is an integer self-replicating system with *p* = 3 and *R* = det(**A**).
4. From our simulations, we observed that for any integer self-replicating system, some of its recurrence matrix **A**’s eigenvalues might be zero and all of other eigenvalues have the same absolute value $\sqrt[{p}]{R}$ where *p* and *R* are some positive integers. Based on this observation, we propose the following hypothesis.
  **Hypothesis**: If any *m* × *m* matrix **A** satisfies all the six conditions above, and also satisfies that there exist positive integers *p* and *R*, and a non-negative and non-zero *m* × 1 vector **N** such that **A**^*p*^ **N** = *R* **N**, then
  $$|\lambda_{1}\left|=\right|\lambda_{2}\left|=\cdots =\right|\lambda_{u}|=\sqrt[{p}]{R}\;\;\text{and}\;\;\lambda_{u+1}=\lambda_{u+2}=\cdots =\lambda_{m}=0$$
  where λ_1_, λ_2_, ⋯, λ_*m*_ are the *m* eigenvalues of **A**, and 1 ≤ *u* ≤ *m*.
  This hypothesis holds numerically for all the cases we have studied, and is a very striking result, but we have been unable to prove it rigorously. We can construct a specific type of graph that satisfies all the six conditions for **A** (each vertex of the graph represents each type of molecule in the system while each edge is assigned the value of the corresponding entry of **A**), and then prove that this type of graph has the properties the hypothesis says. But we have not been able to prove that these conditions for **A** guarantee this type of graph (personal communication with Volodymyr Mazorchuk [22]).

### Population dynamics under the law of mass action

The scheme Eq (5) we applied on analysable self-replicating systems corresponds to a particular population dynamics. However, this population dynamics is inconsistent with most chemical reactions in physical scenarios: Based on the law of mass action, the rate of a chemical reaction is, in general, equal to the arithmetic product of a predefined reaction rate constant and the concentrations of reactants. The rate constant depends on physical properties of the chemicals involved, while the concentrations depend on the physical conditions under which the reaction occurs.

In paper [19], we derived formulas for reaction rates under the law of mass action, in physical scenarios: (1) all molecules are ideally gaseous; (2) the self-replicating system is a well-mixed system and kept in a box under constant pressure and temperature; (3) the resource molecule population in this box is kept constant but finite, achieved by a presumed external unlimited reservoir of resource molecules. Under the law of mass action, for a synthesis reaction $\bar{i}+\bar{j}\to \bar{i+j}$, the reaction rate is (in unit *s*^−1^)

$$\begin{array}{c} \gamma_{+ij}=\omega_{+ij}\cdot N_{i}\cdot N_{j}/N \end{array}$$

where *ω*_+*ij*_ is the rate constant for this synthesis reaction, *N*_*i*_ = *N*_*i*_(*τ*) is the population of molecule $\bar{i}$ in the box at time *τ*, and *N* = *N*(*τ*) is the total population of all the molecules in the box at time *τ*. Note that the variable “time” *τ* is continuous, representing the physical time, which is different from the abstract discrete variable “generation” *t* in the former population dynamics Eq (5). For a decomposition reaction $\bar{i+j}\to \bar{i}+\bar{j}$, the reaction rate is (in unit *s*^−1^)

$$\begin{array}{c} \gamma_{-ij}=\omega_{-ij}\cdot N_{i+j} \end{array}$$

where *ω*_−*ij*_ is the rate constant for this decomposition reaction. The physical conditions and the properties of chemicals assumed in paper [19] guarantee that rate constants *ω*_+*ij*_ or *ω*_−*ij*_ for all reactions in the self-replicating system are identical, thus denoted *ω*. Finally, we use ordinary differential equations (ODEs) to describe its population dynamics.

We now investigate system Eq (3), which is characterised by the golden ratio *ϕ* under the population dynamics Eq (5) we used previously. Here, the mass action population dynamics for this system are

$$\begin{array}{c} \left\{ \begin{array}{c} \begin{array}{cc} dN_{1}/d\tau & =-\omega UN_{1}/N+2\omega N_{2}+\omega N_{3}\\ dN_{2}/d\tau & =-\omega N_{2}+\omega N_{3}+\omega N_{5}\\ dN_{3}/d\tau & =-\omega N_{3}+\omega N_{5}\\ dN_{5}/d\tau & =-\omega N_{5}+\omega UN_{1}/N \end{array} \end{array}\right. \end{array}$$ (15)

where *U* is the constant population of resource molecule $\bar{4}$ in the box, and *N* = *U* + *N*_1_ + *N*_2_ + *N*_3_ + *N*_5_. There are only two parameters. We set *ω* = 1.09986 × 10^11^ *s*^−1^, the same as in paper [19], which is determined by the chemical properties and physical conditions. Note that in physical scenarios, *U* is always finite. We arbitrarily set *U* = 10^10^.

**Fig 3:**
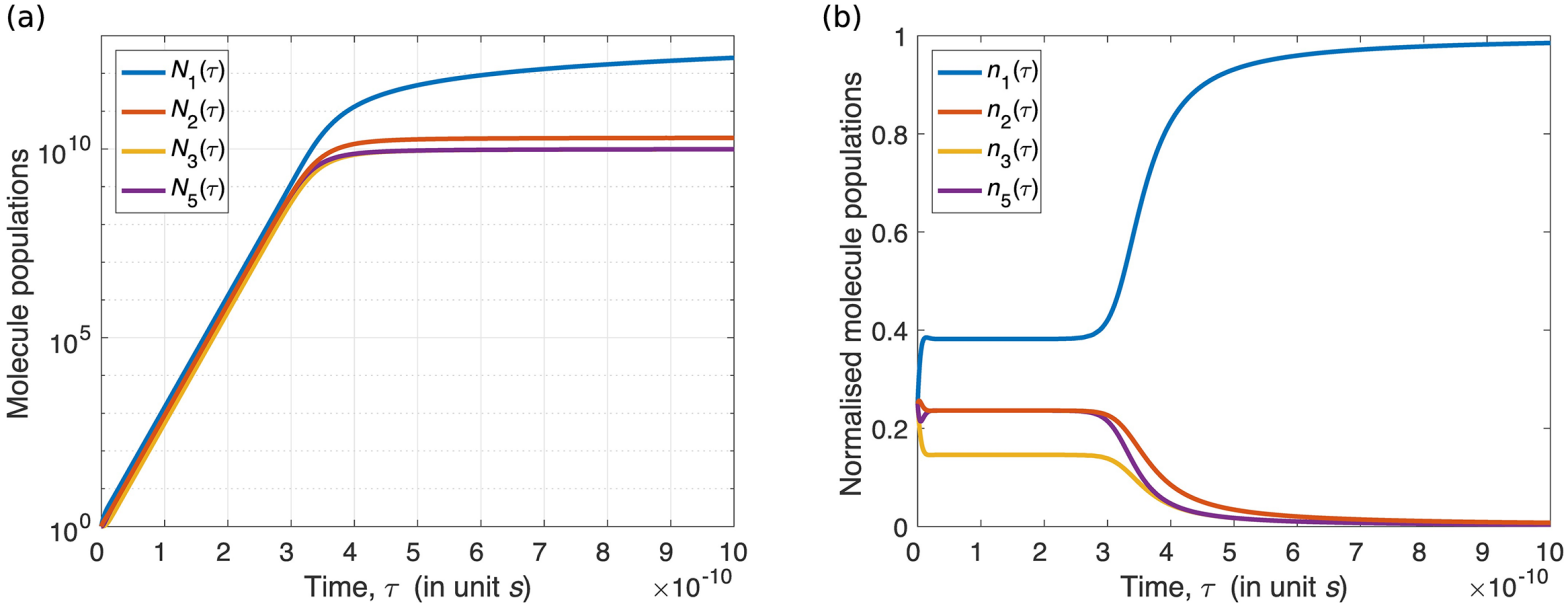
The mass action population dynamics of system Eq (3) in a physical scenario. (a) Solutions of Eq (15) in log-normal scale, i.e., x-axis is in normal scale and y-axis is in logarithmic scale. (b) The normalised molecule populations for the same period as in (a).

Solutions of Eq (15), with initial condition *N*_1_(0) = *N*_2_(0) = *N*_3_(0) = *N*_5_(0) = 1, are shown in Fig 3a, in log-normal scale. After a transient period in the beginning, the populations go into an exponential growth phase (approximately from 0.5 to 2.5 × 10^−10^ *s*). The straight line in log-normal scale implies an exponential growth. After this phase, growing slows down. Fig 3b shows the normalised molecule population *n*_*i*_ = *N*_*i*_/(*N*_1_ + *N*_2_ + *N*_3_ + *N*_5_), to compare with Fig 1. We observe that lim_*τ*→∞_(*N*_1_(*τ*)/*N*_2_(*τ*)) ≡ lim_*τ*→∞_(*n*_1_(*τ*)/*n*_2_(*τ*)) = ∞ which is not *ϕ*.

During the exponential growth phase, however, the normalised population *n*_*i*_ remains almost unchanged (Fig 3b). We further find that *n*_*i*_ during this phase is approximately identical to the corresponding limit value in Fig 1. That is, during the exponential growth phase in Fig 3b, *n*_1_ ≈ 0.38196, *n*_2_ = *n*_5_ ≈ 0.23606, *n*_3_ ≈ 0.14589, and thus *n*_1_/*n*_2_ ≈ *ϕ*. By solving Eq (15) for various values of *U*, we find that the exponential growth phase always occurs, and only occurs when the sum of populations of intermediate molecules is much smaller than population of the resource molecule, namely, *U*/(*U* + *N*_1_ + *N*_2_ + *N*_3_ + *N*_5_) → 1. That is why, in Fig 3a, the exponential growth phase stops around 3 × 10^−10^ *s*, when *N*_1_ + *N*_2_ + *N*_3_ + *N*_5_ becomes of comparable magnitude of *U*. Indeed, if we let *U*/(*U* + *N*_1_ + *N*_2_ + *N*_3_ + *N*_5_) → 1, Eq (15) becomes a linear ODE system, and its solutions are exponential functions. We thus have

$$\begin{array}{c} \lim_{\tau \to \infty }\frac{N_{1}\left(\tau \right)}{N_{2}\left(\tau \right)}=\phi ,\;\;\;\text{if}\;\frac{U}{U+N_{1}+N_{2}+N_{3}+N_{5}}\to 1 \end{array}$$

Therefore, the characteristic number of the system are transient behaviours corresponding to the scenario that all other molecules are much fewer than the resource inside the system, equivalently, the resource inside the system is infinite. It is interesting to note that the Jacobian of the ODEs Eq (15) at the fixed point *N*_1_ = *N*_2_ = *N*_3_ = *N*_5_ = 0 is (**A** − **I**), where **A** is the recurrence matrix for this self-replicating system Eq (3), namely, **A** in Eq (4) (it is in fact a general case that for an analysable self-replicating system, the Jacobian at the zero fixed point of its corresponding ODEs is always (**A** − **I**) where **A** is its corresponding recurrence matrix). In physical scenarios, infinite resource is not possible, so we do not often expect the characteristic number of a self-replicating system to manifest itself.

The transitory nature of characteristic numbers in growth is not the only reason to doubt their universal significance. Note that we also made a strong assumption about the rate constants above that they are all identical, namely, *ω*_*ij*_ = *ω*. As rate constants are determined by the properties of involved chemicals, different reactions are very unlikely to have the same rate constant, in more realistic situations. This observation adds another reason we would not expect *ϕ* to be widely observed in real biological systems.

**Fig 4:**
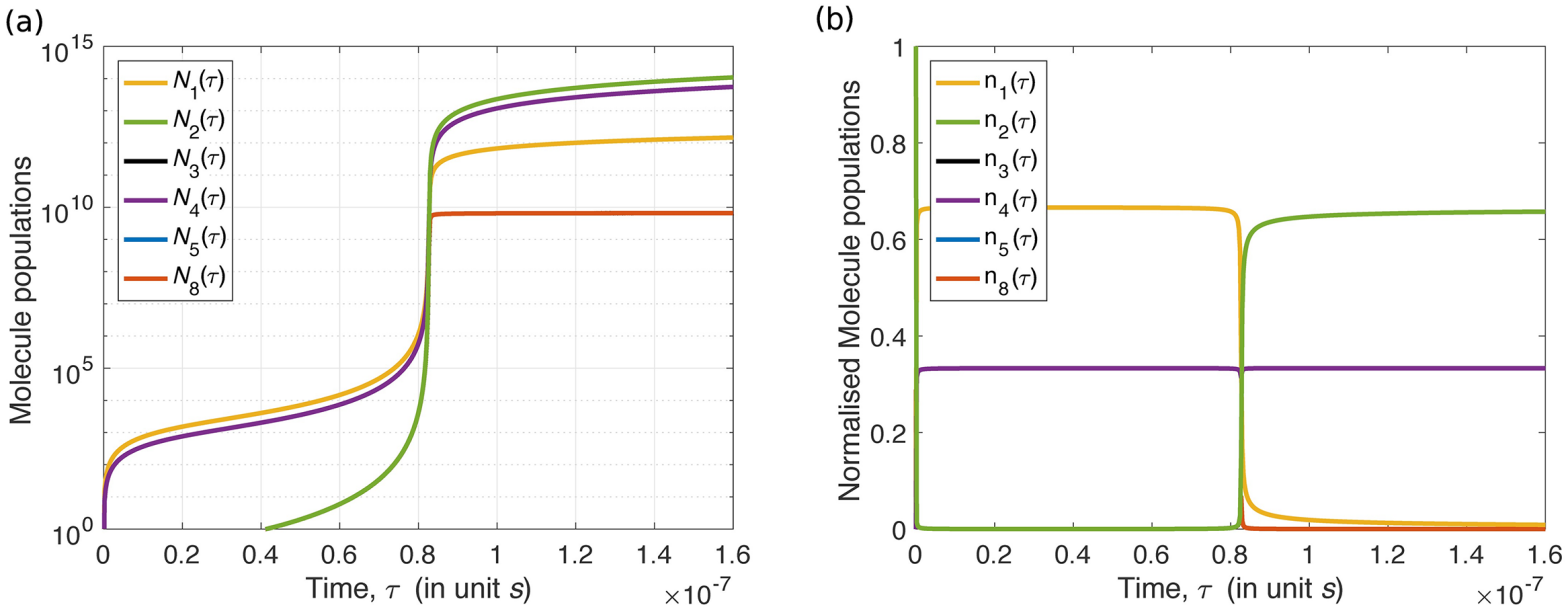
The mass action population dynamics of system Eq (16) in a physical scenario. (a) Solutions of its corresponding ODEs in log-normal scale. Note that populations of molecule $\bar{3}$, $\bar{5}$ and $\bar{8}$ are always the same. (b) The normalised molecule populations for the same period as in (a).

There is yet another factor that limits the generality of the characteristic number of self-replicating systems. Take the following system Eq (16) as an example,

$$\begin{array}{c} \left\{ \begin{array}{c} \begin{array}{cc} \bar{1}+\bar{1} & \to \bar{2}\\ \bar{3} & \to \bar{1}+\bar{2}\\ \bar{5} & \to \bar{1}+\bar{4}\\ \bar{2}+\bar{6} & \to \bar{8}\\ \bar{8} & \to \bar{3}+\bar{5} \end{array} \end{array}\right. \end{array}$$ (16)

where molecule $\bar{6}$ is the resource. Under the mass action population dynamics, this system evolves as in Fig 4. However, it is not analysable by the population dynamics Eq (5): According to criterion 1, 2 and 3 in Section *Materials and methods*, system Eq (16) is a chemically realistic self-replicating system, but intermediate molecule $\bar{1}$ appears twice on the reactant side, which violates criterion 3*. Therefore, it is not analysable by Eq (5), and it thus has no such recurrence matrix. This stricter criterion 3* is essential in allowing us to apply Eq (5). But, it greatly limits the number of systems that we can look at.

The factors listed above, combined with the low number of analysable systems which give rise to *ϕ*, lead us to dismiss the idea that the golden ratio, or any other constant, provides any form of universal characterisation of self-replicating systems.

## Conclusion

A wide range of chemically realistic self-replicating systems can be described by recurrence matrices that can be further characterised by an algebraic number, which is the largest absolute value among all of the eigenvalues of the recurrence matrix. In some cases, the characteristic number is the golden ratio *ϕ*. Yet, *ϕ* is just one of the many numbers that might appear. These could be otherwise an *n*th root of an integer, a generalised golden ratio or certain other algebraic numbers. The characteristic number of a self-replicating system is a transient behaviour, corresponding to the scenario that the resource inside the system is infinite. Moreover, while many systems can be characterised by an algebraic number, there remain many more that cannot be characterised in this way.

Our work suggests that there is no reason to believe that *ϕ* or any other specific number will characterise self-replicating systems. We do not look at data from any specific biological system here, such as ratios of codon frequencies in DNA, but we rather argue that when we study the vast array of all biological systems, then constants such as *ϕ* appear only in a very limited range of situations. It would thus be highly unlikely that the specific, complex self-replicating system that underlies, for example, our DNA is one such system. Our results contradict the claim of some other authors (e.g., in paper [15]) that *ϕ* is universal.

We conclude that (1) *ϕ* has its particular position in chemically realistic self-replicating reaction systems in general but it is not that special since many other constants also appear, and (2) that these constants, including *ϕ*, appear in chemically realistic self-replicating systems only when there is an infinite number of resources inside the system, which is not usually the case in practice. Whenever the golden ratio itself, or some linear function of the golden ratio, is found to characterise the ratio of chemicals in a system, the most likely explanation is that the relationship occurred by chance.

We suggest, instead, that a more useful approach is to develop models that characterise self-replication and investigate their general properties. We see our chemical system and approach as a useful step in this direction.

## Supporting information

S1 Appendix Characteristic polynomial of a matrix. (PDF)
