# Supplementary material for: Is the golden ratio a universal constant for self-replication?
Source: PLoS One. 2018 Jul 16;13(7):e0200601. doi: 10.1371/journal.pone.0200601 (PMC6047800; doi:10.1371/journal.pone.0200601)
Supplement: S1 Appendix — (PDF) [file pone.0200601.s001.pdf]

Supporting Information to:

Is the golden ratio a universal constant for self-replication?

Yu Liu[#*], David Sumpter

Department of Mathematics, Uppsala University, Uppsala, Sweden

[#] Current Address: Molecular Systems Biology, Groningen Biomolecular Sciences and Biotechnology Institute, University of Groningen, Groningen, the Netherlands

[*] Corresponding author
E-mail: yu.ernest.liu@hotmail.com (YL)

Supporting information

S1 Appendix. Characteristic polynomial of a matrix. The characteristic polynomial of an $m \times m$ matrix $\mathbf{A} = (a_{jk})$ is defined as [1]

$$\det(\lambda \mathbf{I} - \mathbf{A}) = \lambda^m - \sigma_1 \lambda^{m-1} + \sigma_2 \lambda^{m-2} - \cdots + (-1)^m \sigma_m$$

where $\mathbf{I}$ is the identity matrix and

$$\sigma_1 = \sum_{j=1}^{m} a_{jj} = trace(\mathbf{A})$$

is the sum of all first-order diagonal minors of $\mathbf{A}$ (equivalently, the trace);

$$\sigma_2 = \sum_{j<k} \det \begin{pmatrix} a_{jj} & a_{jk} \\ a_{kj} & a_{kk} \end{pmatrix}$$

is the sum of all second-order diagonal minors of $\mathbf{A}$;

$$\sigma_3 = \sum_{j<k<l} \det \begin{pmatrix} a_{jj} & a_{jk} & a_{jl} \\ a_{kj} & a_{kk} & a_{kl} \\ a_{lj} & a_{lk} & a_{ll} \end{pmatrix}$$

is the sum of all third-order diagonal minors of $\mathbf{A}$, and so forth. Finally,

$$\sigma_m = \det(\mathbf{A}).$$

References

1. Liesen J, Mehrmann V. In: The Characteristic Polynomial and Eigenvalues of Matrices. Springer International Publishing; 2015. p. 101–113. doi: 10.1007/978-3-319-24346-7_8.
